# Revitalizing the Biochemical Soil Properties of Degraded Coastal Soil Using *Prosopis juliflora* Biochar

**DOI:** 10.3390/life13102098

**Published:** 2023-10-22

**Authors:** Hiba M. Alkharabsheh, Riziki Mwadalu, Benson Mochoge, Benjamin Danga, Muhammad Ali Raza, Mahmoud F. Seleiman, Naeem Khan, Harun Gitari

**Affiliations:** 1 Department of Water Resources and Environmental Management, Faculty of Agricultural Technology, Al Balqa Applied University, Al-Salt 19117, Jordan; 2 Department of Agricultural Science and Technology, School of Agriculture and Environmental Sciences, Kenyatta University, Nairobi P.O. Box 43844-00100, Kenya; 3 Central Highland Eco-Region Research Programme, Kenya Forestry Research Institute, Nairobi P.O. Box 20412-00200, Kenya; 4 National Research Center of Intercropping, The Islamia University of Bahawalpur, Bahawalpur 63100, Pakistan; 5 Institute of Soil Fertilizers and Water-Saving Agriculture, Academy of Agricultural Sciences, Lanzhou 730000, China; 6 Plant Production Department, College of Food and Agriculture Sciences, King Saud University, Riyadh 11451, Saudi Arabia; 7 Agronomy Department, Institute of Food and Agricultural Sciences, University of Florida, Gainesville, FL 32611, USA

**Keywords:** biochar, biochemical properties, cation exchange capacity, nutrients, sub-Saharan Africa

## Abstract

Biochar is an effective soil amendment with capabilities of boosting carbon sequestration and enhancing soil fertility, thus enhancing plant growth and productivity. While numerous studies have documented the positive effects of biochar on improving soil properties, a number of studies have reported conflicting results. Therefore, the current study was conducted to evaluate the impact of *Prosopis juliflora* biochar (0, 2.5, 5.0, and 7.5 t ha^−1^) on soil biochemical properties in Coastal Kenya to ascertain biochar’s potential for soil fertility improvement. A randomized complete block design was used for setting up the experiment with three replicates, while *Casuarina equisetifolia* L. was planted as the test crop. Soil sampling for nutrient analysis was conducted quarterly for 12 months to assess nutrient dynamics under different biochar rates in the current study. Compared to soil untreated with *Prosopis juliflora* biochar, the results showed that there was a significant increase in soil pH by 21% following biochar utilization at the rate of 7.5 t ha^−1^. Total nitrogen was increased by 32% after the biochar application, whereas the total organic carbon was increased by four folds in comparison to biochar-untreated soil. Available phosphorus was increased by 264% following biochar application in comparison to the control treatment. In addition, the application of biochar resulted in an increment in the soil exchangeable cations (Ca^2+^, K^+^, Mg^2+^) across the assessment periods. Soil cation exchange capacity (CEC), bacteria and fungi were enhanced by 95, 33 and 48%, respectively, following biochar application at 7.5 t ha^−1^ in comparison to untreated soil. In conclusion, these results strongly suggest improvement of soil biochemical properties following *Prosopis juliflora* biochar application, thus providing potential for soil fertility improvement in regions such as the one in the study.

## 1. Introduction

Deteriorating soil fertility, due to poor soil management, is the main cause of diminishing land productivity and declining crop productivity [1,2]. The main reason leading to the decline in soil fertility is nutrient depletion through soil erosion and leaching of nutrients such as nitrogen and phosphorus [3,4,5]. Nutrient loss attributed to erosion in soils is estimated to be 45 kg of NPK ha^−1^ yr^−1^ [6,7]. Hence, to enhance crop productivity, there is a need for implementing adaptable measures at the farm level that can solve the problem of diminishing soil fertility [1,3]. Among the many technologies, biochar can offer a cost-effective means of enhancing soil fertility and improving agricultural productivity [8].

Biochar is a by-product of pyrolysis, with particle size diameter ranging from >0.2 mm to <2.0 mm (fine and coarse particles, respectively), rich in carbon, and porous in nature. It is produced through biomass decomposition at low to moderate temperatures (i.e., 300–800 °C) and under a limited oxygen supply [9,10,11]. The most suitable materials for biochar production should contain high lignin concentrations, thus yielding large quantities of biochar [12,13]. Studies have shown that the quality of biochar and its physical and chemical properties are influenced by the production system and source of the feedstock used [14,15,16]. Biochar generated under different pyrolysis temperatures has varying adsorption capacities, mainly resulting from changes in biochar’s surface area during pyrolysis [13,14,17].

The main characteristics of biochar are neutral to high pH, low bulk density, high surface area, porosity, carbon and nutrient contents, and cation exchange capacity (CEC) [18,19,20,21,22]. Such properties enhance biochar’s potential for increasing the productivity of tropical soils [23]. Biochar utilization for enhancing soil fertility is gaining popularity due to its role in improving soil quality, carbon sequestration, and crop yield [24]. Hence, evaluating the chemical and physical properties of biochar is crucial for understanding how it improves soil fertility [25]. The porous and adsorption properties of biochar provide a favorable environment for the growth and reproduction of microorganisms [26]. The increase in pH with biochar application has also been reported to increase bacterial abundance in the soil [27]. Bacteria and fungi populations in the soil are influenced by various factors such as organic matter, porosity, oxygen and carbon dioxide concentration, and soil pH [28].

Biochar’s potential for soil fertility enhancement has been widely explored since the discovery of Terra Preta soils [10]. Numerous studies have investigated biochar’s impact on various soil biochemical properties [21,24,29,30,31]. However, the findings are still inconclusive. The different results reported can be attributed to the usage of biochar produced from diverse feedstocks and at different pyrolysis conditions. The current study utilized *Prosopis juliflora* feedstock for biochar production. *Prosopis juliflora* was introduced in Kenya in the 1970s, among other species from South America, to rehabilitate the Arid and Semi-Arid Areas (ASALs), due to its resilience, fast growth rate, and its many uses, such as for fodder, honey production, shade, windbreak, firewood, building poles, etc. [32,33]. The largest biomass of *Prosopis juliflora* in Kenya is found in the Tana River, Turkana and Baringo counties. Other areas where it is found are Taita Taveta, Kilifi, Samburu, Isiolo, Mandera, Marsabit, Wajir, Kajiado and Migori Counties. It is an invasive species detrimental to livestock and crop production through colonization of critical grazing land, farmlands, and rangelands [29]. However, it is among the leguminous shrubs with some unexploited potential benefits [34,35]. Converting *P. juliflora* to biochar solves two problems: (1) low soil nutrition and (2) the task of eliminating an invasive species that is a threat to local diversity, especially rangelands, and currently has no effective control measures [29,30,36]. 

Given the limited information on the potential of biochar for amelioration of degraded coastal soils, this study was conducted to assess the impact of different *P. juliflora* biochar doses on the biochemical properties of Arenosol in Coastal Kenya. The study was based on the hypothesis that *Prosopis juliflora* biochar can significantly enhance soil biochemical properties.

## 2. Materials and Methods

### 2.1. Study Area

A field experiment was conducted in Gede, Kilifi County, Kenya, situated at 3.2974013° S and 40.001045° E. The average annual rainfall of the region is 800 mm, and the mean temperature is 28 °C. The rainfall in the area is bimodal, with a long rainy season stretching from March to May and a short rainy season that occurs from October to December. The area experiences high evaporation rates ranging from 1800 mm to 2200 mm annually. The dominant soil type in the Gede area is Arenosol, which is characterized by low inherent fertility, sandy texture, and low water-holding capacity [37].

### 2.2. Experimental Design

The experiment was established using a randomized complete block design, having four treatments that were replicated three times. The treatments under investigation were four biochar application rates (0, 2.5, 5.0, and 7.5 t ha^−1^). Biochar was ground into tiny particles (<2.0 mm) to enable proper mixing and incorporation into the uppermost 20 cm soil layer before planting. The plot sizes were 12 m by 12 m, with *Casuarina equisetifolia* L. seedlings of 45 cm height planted in October 2019 as a test crop at a spacing of 2 m by 2 m between and within rows, respectively, as recommended [38]. The biochar applied in the Arenosol field was produced by pyrolyzing the *Prosopis juliflora* feedstock in a continuously running carbonizer at 450 °C for 4 h.

### 2.3. Soil Sampling and Analysis

#### 2.3.1. Soil Sampling

Collection of soil samples was undertaken quarterly for one year, starting from October 2019 at depths of 0–20 cm. Soil sampling was performed with the grid sampling method across the experimental field using a soil auger. Five sampling points (10 cm from the Casuarina trees selected in the grid) for each treatment were used to obtain a composite soil sample. Consequently, samples obtained were placed in zip-lock bags to prevent contamination, then clearly labeled to show treatment, replicate, sampling depth, and date of sampling. The soil samples were transported to the lab in ice-cooled boxes to prevent further dynamic changes. At the onset of the study, soil characterization for physical-biochemical properties was also undertaken. 

#### 2.3.2. Determination of Soil Chemical Properties

Soil pH (2.5:1) (water) and electroconductivity (EC) were measured according to standard procedures as described by Anderson and Ingram [39] and Okalebo et al. [40]. A 20 g soil sample was placed in a 100 mL polythene bottle, followed by the addition of 50 mL deionized water. The contents were shaken for 30 min using a mechanical shaker. The mixture was then allowed to stand for 30 min. The pH (water) was measured using a pH meter (Model 691), and electrical conductivity was measured with a conductivity meter (Model TOA Cm-20S).

Total N was determined using the Kjeldahl method [39,40], where 0.3 g of ground and sieved soil sample was digested using selenium powder and lithium sulphate. The digestion was performed by heating the digestion tubes containing the samples for 2 h at 360 °C. Thereafter, distillation was undertaken using 40% sodium hydroxide. The extract was steamed into 5 mL of 1% boric acid and 4 drops of mixed indicator. Titration was then followed using 0.1 M HCl until the color changed from green through grey to a definite pink. Total nitrogen content in the soil sample was calculated as indicated in Equation (1).

(1) $$Total\;Nitrogen\;content\;in\;the\;soil\;(\%)=\frac{Corrected\;mL\;of\;N/140HCl*0.1}{weight\;of\;sample}$$

where corrected ml of N/140HCl = burette reading − the mL of N/140HCl required for the blank. To obtain the concentration of total nitrogen in g/kg, the percentage total nitrogen was multiplied by 10.

Available phosphorus (P) was determined using the method described by Olsen and Sommers [15] and Okalebo et al. [40], where 5 g of air-dried soil sample was extracted using Olsen’s extracting solution (0.5 M NaHCO_3_) and ascorbic acid reducing agent. The solution was left to stand for 1 h to permit full-color development. Absorbance values of all solutions were measured at 880 nm using a UV spectrophotometer (Model UV Spectronic 21-Milton Roy Co, Markham, ON, Canada). Available P concentrations for each sample were then calculated using Equation (2).

(2) $$P\;in\;soil\;sample\;(ppm)=\frac{C*ppm\;solution*df}{weight\;of\;sample}$$

where C = the corrected concentration of available P in the sample; ppm solution = graph reading; df = dilution factor. 

Extractable potassium (K) was determined spectrophotometrically [39] using a flame photometer, where 5 g of air-dried soil sample was extracted using 1.0M neutral ammonium acetate solution and 27% lanthanum chloride solution. Standards containing potassium at concentrations of 0.0, 2.5, 5.0, 7.5, and 10.0 ppm were prepared similarly to fall within the measurable range of the calibrated flame photometer. The flame emission intensities were measured at 766 nm.

Total organic carbon (TOC) was determined using the Walkey Black method as described by Okalebo et al. [40], where 1.0 g of sieved sample was extracted using 1N K_2_Cr_2_O_7_ (which was prepared by dissolving 49.024 g of dry K_2_Cr_2_O_7_ in 1000 mL distilled water) and concentrated H_2_SO_4_ (98% H_2_SO_4_). Further, the mixture was allowed to stand for 30 min before the addition of 200 mL of deionized water, 1 mL of diphenylamine indicator, and 0.5 g of sodium fluoride. The mixture was back titrated with FeH_8_N_2_O_8_S_2_ solution until the color changed to brilliant green. The volume of the ferrous ammonium sulphate solution consumed was noted. A blank titration (without soil) was also carried out in a similar procedure, and the volume of the ferrous ammonium sulphate solution consumed was noted. Due to the 77% recovery rate of organic carbon in this method, a correction factor of 100/77 (1.3) was used in calculating TOC content (Equation (3)).

(3) $$Total\;organic\;carbon\;(\%)=\frac{\left(B-S\right)*NFAS*0.003*1.3*100}{weight\;of\;sample}$$

where B = volume of ferrous ammonium sulphate consumed for blank titration in ml; S = volume of ferrous ammonium sulfate consumed for sample in ml; NFAS = normality of ferrous ammonium sulfate from blank titration.

Determination of exchangeable Ca, Mg, and Na was performed spectrophotometrically using an atomic absorption spectrophotometer as described by Anderson and Ingram [39], and Okalebo et al. [40]. To obtain a supernatant for analysis, 5 g of soil sample was extracted using 1M ammonium acetate solution of 7.0 pH and concentrated ammonium hydroxide (NH_4_OH). 

Soil CEC was determined by the addition of exchangeable Ca^2+^, Mg^2+^, K^+^, and Na^+^ for pH above 6 using Equation (4).

(4) $$CEC\;({cmol\;kg}^{-1})=\frac{Ca\;(ppm)}{200}+\frac{Mg\;(ppm)}{120}+\frac{K\;(ppm)}{390}+\frac{Na\;(ppm)}{230}$$

#### 2.3.3. Determination of Microbial Population (Bacteria and Fungi)

The determination of microbial population was performed 3 months after the experiment establishment and at the end. Both bacterial and fungal populations in the soil samples were quantified using the dilution plate count method as described by Anderson and Ingram [39]. The growth media for bacteria and fungi contained 39 g of nutrient agar (NA) and 28 g of potato dextrose agar (PDA), respectively, each dissolved in a 1000 mL conical flask of deionized water. For the first dilution, 9 mL of distilled water was measured using a measuring cylinder into test tubes, and 1 g of fresh soil was added and shaken thoroughly. Serial dilution was performed by obtaining 1 mL of the previous water and diluting it with 9 mL of distilled water. The fourth dilution was spread on the NA and PDA, dispensed on Petri dishes (3 replicates per sample). The Petri dishes containing NA were incubated for 12 h in an oven at 37 °C for bacteria to grow, while Petri dishes containing PDA were incubated for 120 h at room temperature to allow fungal growth. A colony counter was then used to count the number of colony-forming units (CFU) in each Petri dish, and the mean was determined for each sample. The number of CFUs in the soil solution was then converted to the number of CFUs per gram of soil.

#### 2.3.4. Baseline Status 

The baseline soil characteristics of the study site and the biochar used for this study are given in Table 1. Soil pH was nearly neutral (6.65), with low levels of EC, TOC, total N, available P, exchangeable K, and exchangeable Ca. Generally, the soils in Kilifi County have low inherent fertility [29,41]. The texture of the soil at the study site was sandy, comprising 90% sand, 6% clay, and 4% silt. The biochar used for the experiment had high pH, calcium, magnesium, potassium, sodium, and total organic carbon contents, whereas its available phosphorus was moderate (26.6 mg kg^−1^).

### 2.4. Statistical Analysis

The data collected during the study were subjected to different statistical analyses. Analysis of variance (one-way ANOVA) was conducted to evaluate the effect of the biochar treatments on the soil biochemical properties, and where significant (*p* < 0.05), means were separated using Tukey’s HSD. Linear regression and Pearson coefficient were conducted to determine the relationships between the variables. The analyses were performed using R software (Version 4.1.0). 

## 3. Results and Discussion

### 3.1. Effect of Biochar Application on Biochemical Soil Properties

#### 3.1.1. Soil pH

Findings of the study show that soil pH differed substantially after utilization of biochar at different dosages (*p* < 0.02, 0.001, 0.0001, and 0.0001 for 3, 6, 9, and 12 months after transplanting (MAT), respectively) (Table 2). Generally, soil pH increased as biochar dosage increased from 2.5 to 7.5 t ha^−1^. Specifically, it increased by 8, 10, and 17% in comparison with the unamended treatment when biochar was applied at 2.5, 5, and 7.5 t ha^−1^, respectively, at 3 MAT. Increases in pH of 9, 14, and 20% compared to unamended control were observed at 6 MAT when biochar application rates of 2.5, 5, and 7.5 t ha^−1^, respectively, were used. A similar increase in soil pH with biochar application was observed at 9 and 12 MAT.

An increase by up to 1.4 pH units was recorded after the utilization of 7.5 t ha^−1^ biochar at 12 MAT when compared to the unamended control. The intermediate rate of biochar (5 t ha^−1^) enhanced pH by 0.97 units in comparison with the unamended treatment at 12 MAT, while the biochar application dose of 2.5 t ha^−1^ enhanced soil pH by 0.54 units above the unamended treatment during the same period. From 3 to 9 MAT, increasing the biochar application rate from 5 to 7.5 t ha^−1^ did not result in significant pH enhancement. Significant pH enhancement with increasing biochar application rates from 2.5 to 7.5 t ha^−1^ was observed at 12 MAT.

**Figure 1:**
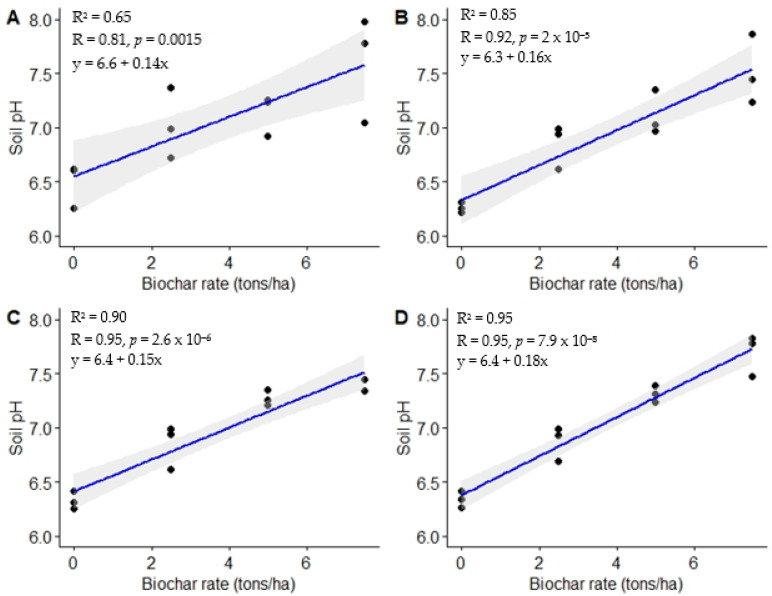
Relationship between biochar application rates and soil pH. (**A**) = 3 MAT, (**B**) = 6 MAT, (**C**) = 9 MAT, (**D**) = 12 MAT.

Strong positive relationships were observed between biochar rates and soil pH at 3, 6, 9, and 12 MAT (r = 0.81, 0.92, 0.95, and 0.97, respectively) (Figure 1). Generally, between 65% to 90% of the changes observed in pH can be attributed to changes in biochar dosage. The highest soil pH (7.69) was observed following utilization of the highest biochar rate (7.5 t ha^−1^), while the unamended treatment resulted in the lowest soil pH (6.34).

The increase in soil pH can be attributed to biochar’s inherent pH, base cation content, calcium carbonate equivalent (CCE), and CaCO_3_ content [42]. The biochar used in the present study had pH of 8.7, which was higher than the soil pH in the study (6.65); this could have led to the increase in soil pH. The free bases in biochar (K, Mg, and Ca) could have been released into the soil solution, thus leading to soil pH enhancement [42]. Soil pH enhancement reported after biochar utilization can also be attributed to an increase in CEC resulting from biochar’s high adsorption capacity [20,43]. Soil pH increase due to biochar application could also result from biochar’s carbonyls (COO^−^), phosphates (PO_4_^3−^), and other alkaline substances that neutralize acidity and enhance soil pH [44].

A comparable study by Hailegnaw et al. [21] reported enhanced soil pH following 8% biochar utilization, with the study concluding that higher biochar rates were more efficient in changing soil properties than lower doses. Similarly, enhanced soil pH (1.6 and 0.8 units) was noted by Mohan et al. [20] and Mwadalu et al. [29], respectively, after biochar application. Geng et al. [44] observed enhanced soil pH of up to 79.25% following the application of woody biochar. Mensah and Frimpong [24] also observed increased soil pH after the usage of corn cob biochar. Zhang et al. [45] and Gao et al. [26] observed enhanced soil pH of 0.5 to 1 unit, respectively. Similarly, Dai et al. [46] reported enhanced soil pH of up to 2.52 units with the utilization of 3% (*v*/*v*) swine manure biochar. 

Biochar’s liming potential is highly dependent on the feedstock used for its preparation. Biochar has been reported to maintain soil pH within the required optimal range for plant development [47,48]. The enhancement of soil pH resulting from biochar application is also influenced by the pyrolysis temperature used to produce the biochar [44]. Biochar’s liming potential is important for the amelioration of acidic soils, thereby enhancing land productivity [20,44,45].

#### 3.1.2. Total Nitrogen

**Figure 2:**
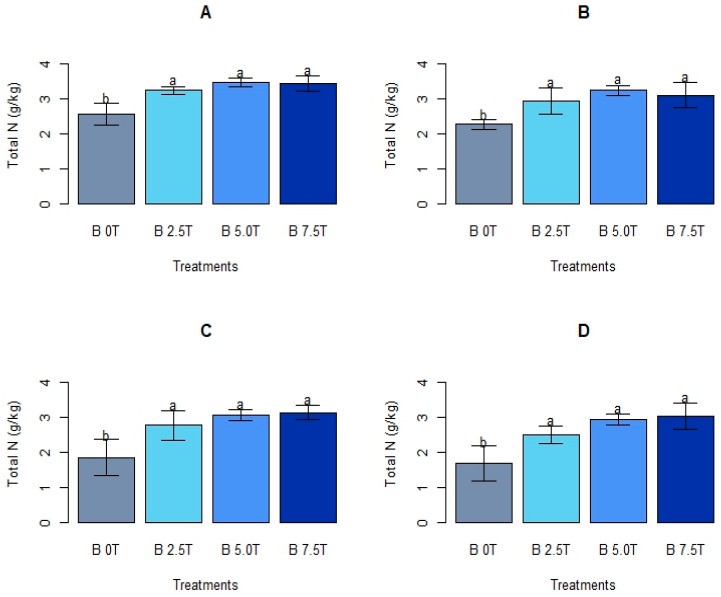
Soil total N under different biochar rates. Means presented with different letters in each graph differ substantially; “B = Biochar”, treatment rates are in t ha^−1^. (**A**) = 3 MAT, (**B**) = 6 MAT, (**C**) = 9 MAT, (**D**) = 12 MAT.

Total N differed significantly after utilization of varying biochar rates (*p* < 0.002, 0.01, 0.008, and 0.02 at 3, 6, 9, and 12 MAT, respectively) (Figure 2). Generally, total N was higher in plots amended with biochar in comparison with the unamended treatment. A biochar dose of 2.5 t ha^−1^ enhanced total N above that of the unamended control by 26.5, 29.1, 33.2, and 47.9% at 3, 6, 9, and 12 MAT, respectively. The intermediate (5.0 t ha^−1^) application rate enhanced total N above that of the control by 35.0, 42.3, 65.9, and 73.3% at 3, 6, 9, and 12 MAT, respectively. The highest biochar dose (7.5 t ha^−1^), on the other hand, enhanced total N above that of the control by 33.5, 36.6, 69.2, and 97.0% during the same assessment periods.

Plots ameliorated with the highest biochar application dose (7.5 t ha^−1^) had the highest total N. Increasing the biochar dose from 2.5 to 7.5 t ha^−1^ did not lead to a significant total N increase during the four assessment periods. However, the three biochar doses used yielded significantly higher total N compared to the unamended treatment. The control plots had the lowest total N across the growth period (Figure 2). 

**Figure 3:**
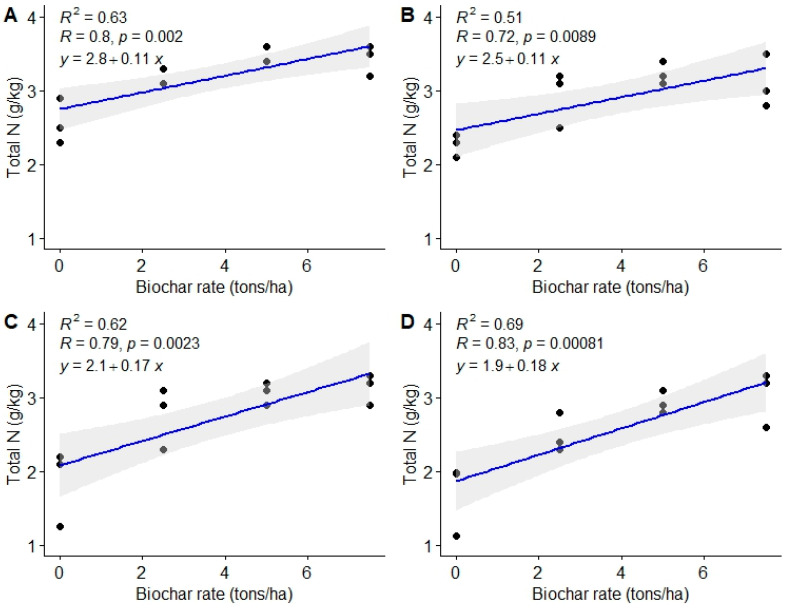
Relationship between biochar doses and soil total N. (**A**) = 3 MAT, (**B**) = 6 MAT, (**C**) = 9 MAT, (**D**) = 12 MAT.

There was a significant positive linear relationship between biochar doses and total N (r = 0.80, 0.72, 0.79, and 0.83) at 3, 6, 9, and 12 MAT, respectively (Figure 3). The present study also revealed a moderate association between biochar rates used and total N (r^2^ = 0.63, 0.57, 0.62, and 0.69), respectively at 3, 6, 9, and 12 MAT, as highlighted in Figure 3. 

Enhanced total N following biochar utilization in the present study can be attributed to N addition from biochar itself. It can also be attributed to the retention of NH_4_^+^ resulting from biochar’s large specific surface area, thus leading to improved N nutrition [24,49]. The increased total N with biochar application could have resulted from a decrease in N leaching due to biochar’s high adsorption capacity [50,51,52].

Mensah and Frimpong [24] reported an increment in the N content when biochar was applied to the soil. Similarly, Zhang et al. [31] reported enhanced total N after biochar application by up to 11.1% in comparison with unamended control, while Hu et al. [49] reported an increase in total N of up to 32.3%. Ghosh et al. [53] attributed N enhancement following biochar utilization to high N content in the biochar used. Rawat et al. [51] observed enhanced soil productivity after biochar utilization through nutrient addition into the soil such as N and retention of nutrients. Phillips et al. [54] reported that the application of biochar, that was produced at 350 °C, resulted in a nitrogen immobilization at the beginning of the experiment, and this could be due to the high carbon content of the biochar. A slight decrease in total N over time was observed; this may be attributed to nutrient uptake (N) by the Casuarina trees planted as a test crop [55]. Nutrient uptake was, however, not quantified in this present study. Similar findings on the decrease in total N with time following biochar utilization were attributed to N uptake and immobilization during the decomposition of biochar [50].

#### 3.1.3. Soil Total Organic Carbon (TOC)

**Figure 4:**
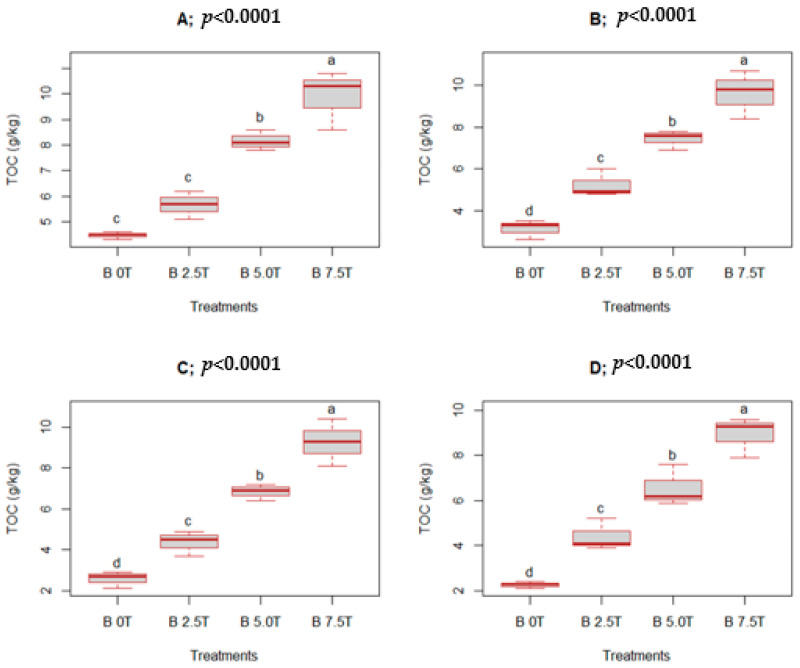
Soil total organic carbon (TOC) under different biochar rates: 3 MAT (**A**), 6 MAT (**B**), 9 MAT (**C**), and 12 MAT (**D**). Means presented with different letters in each boxplot differ substantially; “B” means biochar; treatment rates are in t ha^−1^.

This study shows substantial differences (*p* < 0.0001) in total organic carbon (TOC) following the utilization of varying biochar rates during the four assessment periods: 3, 6, 9, and 12 MAT. Generally, biochar-ameliorated plots had higher TOC compared to the unamended treatment across the assessment periods (Figure 4). The lowest biochar dose (2.5 t ha^−1^) enhanced TOC above that of the control by 26.8, 67.1, 70.0, and 93.8% at 3, 6, 9, and 12 MAT, respectively. On the other hand, the intermediate biochar dose (5.0 t ha^−1^) enhanced TOC by 82.8, 137.4, 165.8, and 189.4% above that of the control at 3, 6, 9, and 12 MAT, respectively. A biochar dose of 7.5 t ha^−1^ (highest dose) enhanced TOC above that of the control by 121.5, 207.7, 260.7, and 293.4% at 3, 6, 9, and 12 MAT, respectively. Increasing the biochar dose from 2.5 to 7.5 t ha^−1^ substantially enhanced TOC (Figure 4).

**Figure 5:**
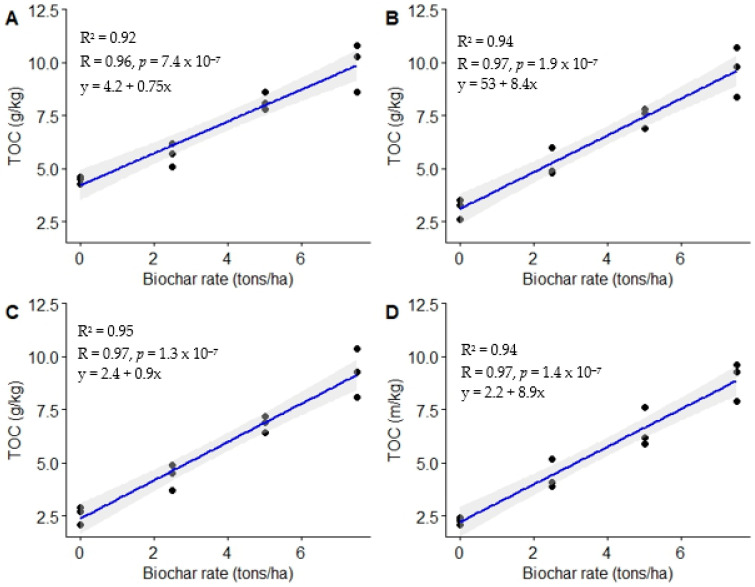
Relationship between biochar doses and total organic carbon. (**A**) = 3 MAT, (**B**) = 6 MAT, (**C**) = 9 MAT, (**D**) = 12 MAT.

Pearson’s correlation analysis showed a strong positive association between biochar doses and TOC at 3, 6, 9, and 12 MAT (r = 0.96, 0.97, 0.97, and 0.97), respectively (Figure 5). The total organic carbon improvement following biochar utilization can be attributed to enhanced organic matter quantities.

Moreover, the enhanced TOC resulting from biochar application is attributed to the high C content (48%) of the biochar used. Biochar contains both labile and recalcitrant C, which significantly enhances TOC when mixed with soil [26,53]. The enhanced TOC resulting from increased biochar doses can be attributed to the increased addition of recalcitrant C. Ghosh et al. [53] reported enhanced TOC following the application of various biochar doses; this is in agreement with the present study (Figure 4). The authors attributed the enhanced TOC to increased biochar quantities added to the soil as biochar doses increased. Similarly, Zheng et al. [22] observed improved TOC after biochar amendment. Equally, Abdullaeva [56] observed a substantial increase in TOC following biochar utilization. Mohan et al. [20] revealed that the application of 3% biochar enhanced organic carbon by 328% above that of the unamended treatment. Gao et al. [26] and Truong and Marschner [27] equally observed enhanced TOC following biochar utilization by ~38%.

Biochar utilization in farming systems is a suitable approach for carbon neutralization ensuing from acting as a carbon sink and reduction of greenhouse gas emissions [8,57]. An increase in soil organic carbon of up to 154% above that of the control after utilization of wheat straw biochar was reported by Ghorbani et al. [57]. Biochar has shown potential for long-term sequestration of carbon [58]. Studies have shown that biochar’s pyrolysis temperature significantly influences carbon sequestration, where higher pyrolysis temperatures produce biochar with a greater capacity for sequestering carbon than biochar produced with lower pyrolysis temperatures [57,59,60,61]. Amending one hectare with biochar sequesters approximately 13 tons of CO_2_eq in the soil [62]. During pyrolysis, 0.04 tCO_2_eq is emitted to the atmosphere for each ton of biomass pyrolyzed; however, 1.67 tCO_2_eq per ton of feedstock is stored in the soil. Biochar captures and stores carbon in recalcitrant form, thereby reducing carbon emissions into the atmosphere [63]. Biochar use in agriculture is a feasible option for enhancing carbon sequestration and improving soil quality [59,64]. The recalcitrant nature of biochar is important for its application in carbon capture and sequestration, as highlighted by Zhang et al. [59]. Post-Kyoto agreements under the United Nations Framework Convention on Climate Change (UNFCCC) unilaterally accepted biochar as a viable strategy for mitigating climate change [65].

Total organic carbon declined across the assessment period for all treatments, with the control recording a decrease of up to 50%, while a biochar dose of 7.5 t ha^−1^ yielded a decline in TOC of up to 10%. Studies have shown that the decomposition of biochar ranged from 0.005% to 0.023% per day, with the decomposition rate reducing as biochar doses increased [66]. Biochar decreases decomposition rates of organic matter to facilitate C sequestration in low organic C soils [67,68].

#### 3.1.4. Available Phosphorus

Substantial available P differences were observed after utilization of varying biochar rates (*p* < 0.001, 0.0009, 0.0001, and 0.0001 at 3, 6, 9, and 12 MAT, respectively). Generally, biochar-amended plots had higher available P than the control (Table 3). Biochar dosage of 7.5 t ha^−1^ led to higher available P levels across the assessment periods, by 264, 302, 282, and 264% compared to the control at 3, 6, 9, and 12 MAT, respectively. The intermediate application rate (5.0 t ha^−1^) enhanced available P above that of the control by 194, 238, 231, and 176% at 3, 6, 9, and 12 MAT, respectively. On the other hand, the lowest biochar dose enhanced available P above that of the control by 152, 184, 151, and 90% at 3, 6, 9, and 12 MAT, respectively. Increasing the biochar dose from 5.0 to 7.5 t ha^−1^ did not significantly increase available P from 3 to 9 MAT (*p* > 0.05). However, substantial variations in available P were observed between biochar doses of 2.5 and 7.5 t ha^−1^. 

**Figure 6:**
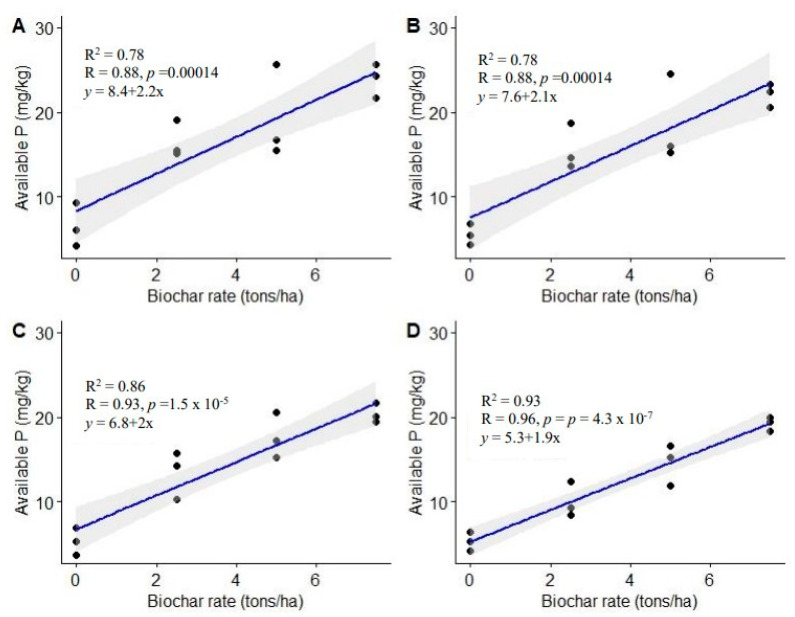
Relationship between biochar application rates and soil available P. (**A**) = 3 MAT, (**B**) = 6 MAT, (**C**) = 9 MAT, (**D**) = 12 MAT.

Results of Pearson’s correlation revealed a positive linear relationship between biochar applied and available soil P, of r = 0.88, 0.88, 0.93, and 0.96 at 3, 6, 9, and 12 MAT, respectively (Figure 6). The study further revealed that 78 to 93% of the changes observed in available P could be attributed to the biochar doses used.

The increase in available P following biochar utilization as observed in this study can be attributed to P addition through biochar application. The enhanced available P could as well be attributed to reduced aluminum (Al) and iron (Fe) activity as a result of increased pH and exchangeable bases [24,69]. Biochar’s ability to retain and exchange phosphate ions resulting from its positively charged surface also could have contributed to improved available P in the present study [51].

Similar observations on enhanced available P with biochar application were made by Mensah and Frimpong [24]. Biochar utilization substantially improved soil P, with the highest P observed in the 2% sole biochar application. Ghosh et al. [53] equally observed improved available P in biochar-amended plots. Soil P has been reported to be highly pH-dependent [70]. Studies by NRCS [70] and Jensen [71] reported that available P is mainly available between pH levels of 6.0 and 7.5. The enhanced available P resulting from increasing biochar doses can thus be linked to the liming effect of biochar, which was enhanced when biochar rates were increased in the present study.

Glaser and Lehr [72] reported improved available P with increased biochar application rates, a finding that is also reported in the current study. Biochar can potentially serve as sustainable P fertilizer, with its impact depending on pyrolysis temperature, feedstock, and application doses [72]. Woody biochar has higher P than biochar from manure [71,73,74]. Biochar produced from high temperatures (>550 °C) has a larger surface area and P content [73]. Generally, the P concentration steadily increases as pyrolysis temperature increases [72,73]. A study by Li et al. [73]) reported enhanced P during the pyrolysis process by two to three folds.

Approximately 50% of phosphorus from biochar is released in the form of orthophosphates and pyrophosphates at pH < 9 [73]. Phosphorus adsorbed in biochar surfaces is often released as part of biochar’s aging process and utilized by plants. Enhanced P with biochar application contributes to greater root growth, thus enhancing nutrient and water uptake by plants [69,73]. There was also a slight decline in available P across the assessment periods in the current study. The decline could have resulted from P uptake by the test crop (Casuarina trees). However, such nutrient uptake of Casuarina trees in the present study was not quantified. Liao et al. [75] reported similar findings on the decrease in soil P with time and attributed the decline to nutrient uptake by maize and faba bean during a three-year experiment period.

#### 3.1.5. Extractable Potassium

The findings of this study show significant (*p* < 0.001) variation in extractable K across the four assessment periods (Table 4) following the utilization of different biochar doses. Generally, biochar-ameliorated plots recorded higher extractable K compared to the untreated control. For instance, the highest biochar dose (7.5 t ha^−1^) led to the highest extractable K levels across the assessment periods, by 102, 107, 126, and 123% compared to the control at 3, 6, 9, and 12 MAT, respectively. The intermediate application rate (5.0 t ha^−1^) enhanced extractable K above that of the control by 85, 77, 94, and 90% at 3, 6, 9, and 12 MAT, respectively, whereas the respective increases in K associated with the lowest biochar application rate were 41, 31, 35, and 47% at 3, 6, 9, and 12 MAT, respectively. There was, however, no significant difference in extractable K between biochar doses of 5.0 and 7.5 t ha^−1^ across the four assessment periods (*p* > 0.05). The general trend in the present study was control < 2.5 t ha^−1^ < 5.0 t ha^−1^ = 7.5 t ha^−1^, except at 9 MAT, where the trend was control = 2.5 t ha^−1^ < 5.0 t ha^−1^ = 7.5 t ha^−1^.

**Figure 7:**
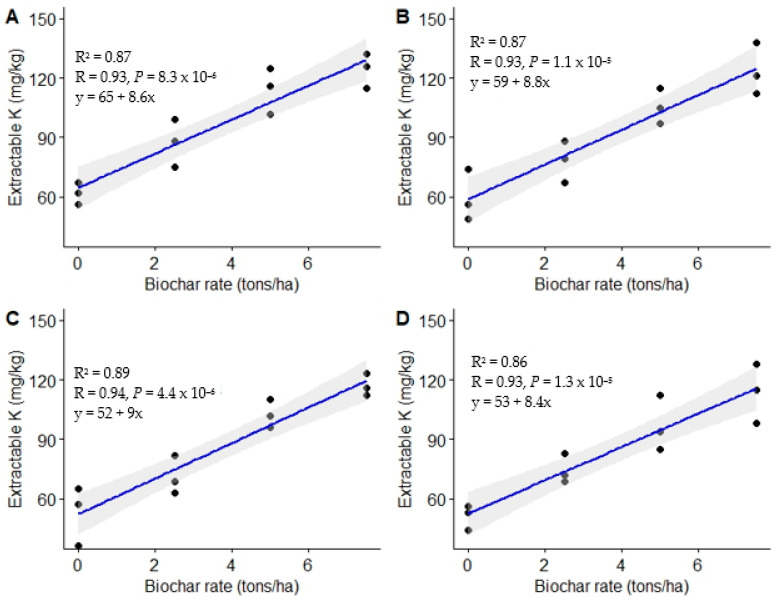
Relationship between biochar rates and extractable K. (**A**) = 3 MAT, (**B**) = 6 MAT, (**C**) = 9 MAT, (**D**) = 12 MAT.

Significant positive linear relationships between biochar rates and extractable K were observed across the four assessment periods (r = 0.93–0.94) (Figure 7). This was an implication that 86 and 89% of the observations made in extractable K could be attributed to the response to changes made in biochar dosages (Figure 7).

The enhanced extractable K that resulted from different biochar application rates in the present study could be attributed to the use of biochar, which was rich in the element (1166 mg kg^−1^). Enhanced extractable K following biochar utilization may have also resulted from increased K-solubilizing bacteria [76]. In addition, the improved extractable K in the current study could have resulted from the ash content in the biochar used for the experiment (Mensah and Frimpong, [24]); the biochar used for the experiment had an ash content of 3.95% (*w*/*w*). The enhanced K^+^ could also be ascribed to the decreased leaching of the element and its release as adsorbed K^+^ to the soil solution due to biochar’s large surface area and high adsorption capacity. K^+^ availability in the soil is pH-dependent, with high pH enhancing its availability [77,78,79]. Miller [78] reported that K^+^ availability is higher above a pH of 6.0. This could explain the higher K^+^ when biochar was utilized, which substantially raised soil pH.

A comparative study by Gao et al. [26] reported enhanced extractable K of up to 22% following biochar application. Similar findings on improved extractable K after biochar utilization were also reported by Ghosh et al. [53] and Wang et al. [79]. Syuhada et al. [50] equally observed a substantial impact of biochar doses on K availability, with the utilization of biochar at 10 to 15 g kg^−1^ leading to substantially higher K than that in the untreated control. Jien and Wang [80] also observed a significantly enhanced K following biochar utilization. Dume et al. [77] observed improved exchangeable K^+^ by up to 73% following biochar application. The study attributed the changes in K^+^ following biochar utilization to the ash content present in the biochar. There was also a slight decline in extractable K across the assessment periods; which may have resulted from nutrient uptake by Casuarina trees planted as test crops [55].

#### 3.1.6. Cation Exchange Capacity (CEC)

Results of the present study show substantial variations in CEC following the utilization of varying biochar doses at 9 and 12 MAT (*p* < 0.05 and *p* < 0.02, respectively) (Table 5). The CEC was not significantly different at the onset of the experiment (3 and 6 MAT). There was, however, a general increase in soil CEC after biochar utilization. A biochar dose of 7.5 t ha^−1^ yielded the highest CEC (Table 5). Cation exchange capacity was enhanced by 39% and 95% with the utilization of the biochar dose of 7.5 t ha^−1^ at 3 and 12 MAT, respectively. The general trend of CEC in the soil in the present study at 3 and 6 MAT was control = 2.5 t ha^−1^ = 5.0 t ha^−1^ = 7.5 t ha^−1^, while at 9 and 12 MAT, the trend was control < 2.5 t ha^−1^ = 5.0 t ha^−1^ < 7.5 t ha^−1^.

**Figure 8:**
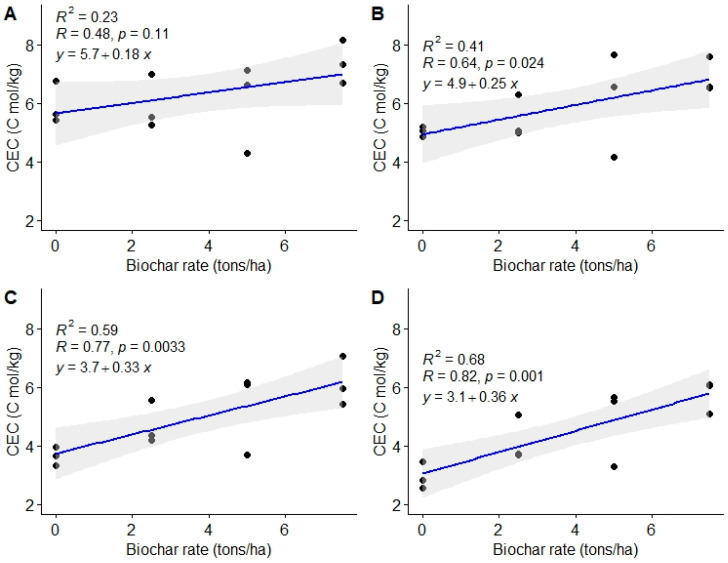
Relationship between soil biochar doses and soil cation exchange capacity (CEC). (**A**) = 3 MAT, (**B**) = 6 MAT, (**C**) = 9 MAT, (**D**) = 12 MAT.

The study also revealed a substantial positive linear relationship between biochar doses and soil CEC (Figure 8). The Pearson’s correlation coefficient for the four assessment periods of 3, 6, 9, and 12 MAT were r = 0.48, 0.64, 0.77, and 0.82, respectively.

The high CEC observed after biochar amendment may have been due to the addition of exchangeable cations through biochar application [51]. The enhanced CEC resulting from biochar application can also be attributed to low biochar oxidation, which increases char’s carboxyl groups, thus improving soil CEC [16,20,78].

Soil CEC is an important soil quality index that determines the soil’s capacity to supply nutrients to plants [51]. It measures the capacity of the negative charge where cationic nutrients are adsorbed by the soil. Comparable studies have reported increased CEC after biochar utilization [24,26,27]. Mohan et al. [20] reported enhanced Na^+^, K^+^, Ca^2+,^ and Mg^2+^ availability after biochar use. The study further reported an increase in CEC of up to 362% at higher biochar application rates in comparison with the unamended control. Kaur and Sharma [81] reported CEC increases of up to 33.2% following biochar application. This is contrary to the findings by Abdullaeva [56], where no substantial increase in CEC after biochar application was observed.

The relatively high CEC values attributed to biochar application are an indication of its capacity to retain soil nutrients [81]. Studies have shown that CEC is also pH dependent whereby higher pH results in higher CEC [82]. This could explain the enhanced CEC after biochar utilization in the present study due to biochar’s liming properties, which significantly increased soil pH. Biochar’s effect on CEC is dependent on the feedstock used; feedstock from animal waste has higher CEC than plant wastes [81]. De melo Carvalho et al. [8] observed a linear increase in soil pH, exchangeable Ca, exchangeable Mg, and CEC after biochar utilization. The findings of the present study are contrary to the findings by Abdullaeva [56] that reported no substantial CEC improvement as a result of biochar utilization. 

### 3.2. Effect of Biochar on Soil Microbial Population

Findings of the present study show substantial variations in bacterial (*p* < 0.02 and 0.01 at 3 and 12 MAT, respectively) and fungal populations (*p* < 0.01 and 0.02 at 3 and 12 MAT, respectively) in the soil following utilization of varying biochar rates (Table 6). The soil had a higher population of bacteria than of fungi. Generally, plots ameliorated with biochar yielded a higher microbial population (bacteria and fungi) than the unamended control.

The lowest biochar dose of 2.5 t ha^−1^ enhanced the bacterial population above that of the control by 13 and 16% at 3 and 12 MAT, respectively, and the fungal population by respective magnitudes of 17 and 21% during the two assessment periods. The intermediate biochar rate (5.0 t ha^−1^) enhanced the bacterial population above that of the control by 23 and 22% at 3 and 12 MAT, respectively. It further enhanced the fungal population by 33 and 45% during the two assessment periods, respectively. The highest biochar application rate (7.5 t ha^−1^) yielded the highest bacterial population, above that of the control by 33% at both 3 and 12 MAT. It also enhanced the fungal population above that of the untreated control by 45 and 48% for the two assessment periods, respectively.

Generally, there was a slight rise in the microbial population by <5.6% from 3 MAT to 12 MAT resulting from biochar application. However, increasing the biochar dose from 2.5 to 7.5 t ha^−1^ did not lead to a substantial improvement in the microbial population (Table 6). The general trend of the bacterial population at 12 MAT was: control < 2.5 t ha^−1^ = 5.0 t ha^−1^ = 7.5 t ha^−1^_,_ while that of the fungal population was control = 2.5 t ha^−1^ < 5.0 t ha^−1^ = 7.5 t ha^−1^.

The increase in soil microbial population after biochar utilization could be due to biochar’s large surface area and porosity, which provide a habitat for soil microorganisms [83]. The high bacterial population in biochar-ameliorated plots could also be attributed to enhanced soil organic matter, which improved aeration and porosity, thus providing a conducive habitat for microorganisms [84]. Gul et al. [85] noted that biochar’s porosity and large surface area serve as a habitat for bacteria and fungi. The study further revealed that the population of bacteria and fungi was influenced by biochar’s surface charge, which binds chemical compounds, microbial cells, ions, and concentrations of nutrients.

Ullah et al. [86] observed enhanced soil bacteria of up to 16% following the utilization of 2% biochar. Increasing the application rate to 4% increased the bacterial population by up to 50%. Azeem et al. [87] revealed that biochar can promote plant development resulting from improved microbial activity in the soil through its impact on microbial abundance, bacteria/fungi ratio, and microbial community structure. Zhang et al. [88,89] and Zhao et al. [90] similarly observed enhanced soil microbial biomass of up to 763% with biochar application.

## 4. Conclusions

The findings of this study show that *Prosopis juliflora* biochar as a soil amendment can significantly improve the soil biochemical properties of degraded Arenosol. In particular, *Prosopis juliflora* biochar at the rate of 7.5 t ha^−1^ enhanced the soil pH, total N, TOC, and CEC, thereby improving P availability. The improvement in soil nutrients following *Prosopis juliflora* biochar utilization can be crucial for enhancing plant growth and development. In addition, *Prosopis juliflora* biochar increased the bacterial and fungal populations, which can be crucial for organic matter mineralization. The study further showed that higher biochar rates offered optimal benefits in terms of enhancing soil biochemical properties. Further studies are recommended on the effects of biochar produced from other tree species on soil properties in such regions.

## Figures and Tables

**Table 1:** Baseline characteristics of site and biochar used for the experiments.

Parameter	Site Characteristics	Biochar Characteristics
pH	6.65	8.7
EC (mS cm^−1^)	0.052	1.39
Total N (g kg^−1^)	1.60	8.40
Total organic C (g kg^−1^)	3.40	480.0
Exchangeable K (mg kg^−1^)	62	1166.1
Available P (mg kg^−1^)	6.8	26.6
Exchangeable Na (mg kg^−1^)	-	989
Exchangeable Ca (mg kg^−1^)	200	746
Exchangeable Mg (mg kg^−1^)	149	354
Ash (g kg^−1^)	-	39.5
CEC (cmol kg^−1^)	-	11.2

**Table 2:** Soil pH under different biochar rates.

Parameter	pH ± SE			
Treatments	3 MAT	6 MAT	9 MAT	12 MAT
B 0T	6.50 ± 0.21 b	6.26 ± 0.04 c	6.33 ± 0.08 c	6.34 ± 0.08 d
B 2.5T	7.02 ± 0.33 ab	6.85 ± 0.20 b	6.85 ± 0.20 b	6.87 ± 0.16 c
B 5.0T	7.14 ± 0.19 a	7.12 ± 0.20 ab	7.27 ± 0.07 a	7.31 ± 0.07 b
B 7.5T	7.60 ± 0.50 a	7.52 ± 0.32 a	7.41 ± 0.06 a	7.69 ± 0.18 a
*f* _(3,8)_	5.83	17.83	50.83	56.38
*p* value	0.02	0.001	0.0001	0.0001

Means presented with different letters in each column differ significantly. MAT denotes months after transplanting. Treatments B 0, B 2.5, B 5.0, and B 7.5 denote different rates of applied biochar: 0, 2.5, 5.0, and 7.5 t ha^−1^, respectively.

**Table 3:** Available P under different biochar rates.

Treatments	Available Phosphorus ± SE (mg kg^−1^)			
	3 MAT	6 MAT	9 MAT	12 MAT
B 0T	6.56 ± 2.54 c	5.51 ± 1.23 c	5.35 ± 1.58 c	5.30 ± 1.10 d
B 2.5T	16.56 ± 2.17 b	15.67 ± 2.72 b	13.44 ± 2.79 b	10.05 ± 2.08 c
B 5.0T	19.29 ± 5.49 ab	18.62 ± 5.19 ab	17.69 ± 2.70 a	14.60 ± 2.45 b
B 7.5T	23.85 ± 1.89 a	22.13 ± 1.42 a	20.41 ± 1.52 a	19.27 ± 0.83 a
*f* _(3,8)_	14.13	16.20	27.54	35.42
*p* < 0.05	0.001	0.0009	0.0001	0.0001

Means presented with different letters in each column differ significantly. Treatment B 0, B 2.5, B 5.0, and B 7.5 denote different rates of applied biochar: 0, 2.5, 5.0, and 7.5 t ha^−1^, respectively.

**Table 4:** Extractable K under different biochar doses.

Treatments	Extractable Potassium ± SE (mg kg^−1^)			
	3 MAT	6 MAT	9 MAT	12 MAT
B 0T	61.7 ± 5.5 c	59.7 ± 12.9 c	52.7 ± 15.0 b	51.0 ± 6.3 c
B 2.5T	87.3 ± 12.0 b	78.0 ± 10.5 b	71.3 ± 9.7 b	74.7 ± 7.4 b
B 5.0T	114.3 ± 11.6 a	105.7 ± 9.0 a	102.0 ± 7.0 a	97.0 ± 13.8 a
B 7.5T	124.3 ± 8.6 a	123.7 ± 13.2 a	117.0 ± 5.6 a	113.7 ± 15.0 a
*f* _(3,8)_	24.93	18.24	25.71	17.50
*p* < 0.05	0.0002	0.0006	0.0002	0.0007

Note: Means presented with different letters in each column differ significantly. Treatment B0, B2.5, B5.0, and B7.5 denote different rates of applied biochar: 0, 2.5, 5.0, and 7.5 t ha^−1^, respectively.

**Table 5:** Soil cation exchange capacity under different biochar application rates.

Treatments	CEC ± SE (cmol kg^−1^)			
	3 MAT	6 MAT	9 MAT	12 MAT
B 0T	5.93 ± 0.72 a	5.03 ± 0.17 a	3.65 ± 0.32 b	2.95 ± 0.46 b
B 2.5T	5.94 ± 0.93 a	5.45 ± 0.74 a	4.70 ± 0.74 ab	4.16 ± 0.78 ab
B 5.0T	6.03 ± 1.51 a	6.13 ± 1.79 a	5.33 ± 1.40 ab	4.84 ± 1.33 a
B 7.5T	8.17 ± 2.04 a	7.60 ± 1.84 a	6.15 ± 0.85 a	5.76 ± 0.57 a
*f* _(3,8)_	1.87	2.12	3.99	5.77
*p* < 0.05	0.21	0.17	0.05	0.02

Note: Means presented with different letters in each column differ significantly. Treatment B0, B2.5, B5.0, and B7.5 denote different rates of applied biochar: 0, 2.5, 5.0, and 7.5 t ha^−1^, respectively.

**Table 6:** Bacterial and fungal populations under different biochar rates and assessment periods.

Treatments	CFUs g Soil^−1^			
	Bacteria × 10^5^		Fungi × 10^4^	
	3 MAT	12 MAT	3 MAT	12 MAT
B 0T	48 ± 4.9 b	49 ± 4.0 b	42 ± 8.1 c	42 ± 8.6 b
B 2.5T	54 ± 6.6 bc	57 ± 2.6 a	49 ± 4.0 bc	51 ± 4.6 ab
B 5.0T	59 ± 4.2 ab	60 ± 5.5 a	56 ± 5.5 ab	55 ± 5.7 a
B 7.5T	64 ± 4.5 a	65 ± 6.1 a	61 ± 4.7 a	62 ± 4.0 a
*f* _(3,8)_	5.91	6.64	6.80	5.58
*p* < 0.05	0.02	0.01	0.01	0.02

Note: Means presented with different letters in each column differ significantly. Treatment B 0, B 2.5, B 5.0, and B 7.5 denote different rates of applied biochar: 0, 2.5, 5.0, and 7.5 t ha^−1^, respectively.

## Data Availability

All data generated or analyzed during this study are included in this article.
